# Progress in Surgery

**Published:** 1897-07-24

**Authors:** 


### Progress in Surgery.

### RHINOLOGY.

*(Concluded from page 270.)*

**Ethmoidal Sinus.**—A consideration of the anatomical relations of the sphenoidal sinuses would lead one to expect loss of vision from suppuration within them. A case in point is related by H. F. Hansell,[11] of Philadelphia. A lad, aged seventeen, awoke one morning with severe frontal headache and an almost absolute loss of vision. He had gone to bed the night before in his usual health, free from any disturbance of sight. During the week following the onset of the attack his vision became worse and his mental powers somewhat dulled, but the excruciating headache passed off. On examination of the eye, dilated pupils which were unresponsive to light, punctate hyalitis, and œdema of the retina with detachment towards the periphery were detected. In the absence of an assignable cause for the ocular and cerebral œdema the condition of the nose was investigated. It was found to be completely blocked anteriorly by greatly swollen turbinals, while posteriorly muco-pus flowed from the choanæ. On further examination pus was observed to issue from the superior and middle meatuses of both sides, and from the upper and back part of the left fossa; the maxillary and frontal sinuses proved to be normal. The disease was diagnosed as acute purulent inflammation of the anterior and posterior ethmoidal cells on both sides, and of the left sphenoidal sinus. Atropine internally, with appropriate local treatment of the intra-nasal congestion, quickly caused the inflammation to subside. Notwithstanding the nasal treatment whereby a cure of the ethmoidal and sphenoidal disease was effected, "the improvement in vision has been slight. The fields have gradually enlarged and now include the unbroken periphery, in which light projection has returned, and the fingers can be counted on the temporal sides, but a large central scotoma persists. The pupils have become responsive to light. The optic nerves have markedly atrophied and the retinæ degenerated." Hansell thinks the least unsatisfactory explanation of the ocular complication is that of a localised meningitis, accompanied by marked swellings and œdema of the periosteum covering the body of the sphenoid, induced by purulent disease of its cells through contiguity of tissue.

**Nasal Obstruction.**—The cardiac symptoms referable to nasal obstruction are discussed by G. R. Lockwood.[12] In concluding his paper he summarizes his remarks as follows: 1. It is highly probable that patients with cardiac disease are more subject than others are to nasal obstruction. 2. Nasal obstruction occurring in a patient with cardiac disease may upset the balance of respiratory compensation and produce decided symptoms. 3. Unless care be taken, these symptoms may be mistaken for those of failing compensation, and may lead to a gloomy prognosis and a faulty treatment. 4. Unless the nasal obstruction be properly relieved and the patient allowed a sufficient quantity of good air, the arterial spasm may possibly occur, throwing an increased amount of work on the heart already handicapped, and may become a factor in inducing dilatation. The effect of the poor quality of the blood thus supplied to the endocardium must also be taken into consideration. 5. Nasal examination made during the day may not reveal the actual obstruction. In fact the most characteristic cases are those in which the patient is waked at night with dyspnœa. He may be well during the day, or may complain only of moderate shortness of breath. Examination of the nose during the day may reveal abundance of air space. Usually, however, the turbinated bodies show great vasomotor irritability, and rapidly swell when they are irritated by a probe, so as to occlude the air-passages. The nocturnal attacks are characterized by dyspnœa, præcordial distress, feebleness and irritability of the heart's action, and a sense of impending death. Immobility of the thorax during an attack does not, however, seem to occur. The attacks appear to be caused by asphyxia, due to congestion and swelling of the posterior portion of the turbinated bodies. This congestion is favoured by the recumbent position of the patient and by the sluggish state of the circulation, especially during the early morning hours. The dyspnœa, præcordial pain and distress, intermittency of the heart, and subjective palpitation seem to be evidences of arterial spasm from the irritation of suboxidised products within them. Similar congestive states may exist during the day, but the patient is then awake, and able to breathe by preference through the mouth so as to get plenty of air.

**Diseases of the Septum.**—Gougenheim[13] writes on traumatism of the septum. He remarks that traumatism of the nose may result in two kinds of lesions, viz., fracture or dislocation; but these lesions, which usually are simple, may also be complicated by hæmatoma, or abscess of the septum. The vomer, situated far back, is rarely fractured, but the more anterior cartilaginous septum is liable not only to be bent, but to be fractured or dislocated, and the latter accident, as has been shown by Mollière, of Lyons, is " extremely frequent."

Among complications of these fractures are liquid tumours of the septum. These tumours, which almost invariably are the result of traumatism, may contain pus, a serous fluid, or blood. A serous fluid is only met with in long-standing hæmatomata, in which absorption has commenced. With regard to pus, it may form at once, inasmuch as there may be communication between the fracture and the outer air, giving rise to infection. It has been alleged, however, that the tumour at first invariably contains blood, and that pus only makes its appearance secondarily. Whether containing blood or pus, these tumours present the same appearance. They are situated at the entrance to the nasal cavities, which they almost entirely obstruct, and are covered by the mucous membrane, which is but slightly altered at this point and retains its pink colour. These tumours almost invariably result from a fracture. Occasionally, however, an abscess may be met with which does not appear to be caused by a fracture. The duration of these abscesses varies according to the nature of the tumour. If this only contains blood, the pain is moderate, and there is no fever, but only slight discomfort and obstruction of the nose, the tumour, therefore, being well borne. If, on the contrary, it contains pus, there is considerable fever and severe pain, inducing the patient to seek medical advice at once. This refers, of course, only to acute, and not to chronic abscesses, which follow osseous lesions of tuberculous or syphilitic nature, and are not usually consecutive to traumatism. The abscess, as a rule, is bilateral, cases in which it occupies only one side being extremely rare. Usually it has the form of a double sac, the two parts of which are in communication through the fracture; but double abscesses, not communicating with each other, are occasionally met with.

Septal deviations and their treatment have recently been treated at some length in these columns, but we must refer to Griffin's[14] method of treatment of this often troublesome condition. He remarks that he has had one case of deflection in a boy of five, resulting from a foreign body which formed the nucleus of a rhinolith, and another very similar case in a girl of six. Zuckerkandl states that he always found in his researches that the septum was straight before the seventh year.

Griffin remarks that a great deal of the success of the operation depends upon the condition the mucous membrane is in when the operation is attempted. He gives five to ten grains of quinine at bed-time for a week before operating, and has found that this greatly facilitates the removal of any congestion that may have involved the mucous membrane and at the same time reduces the hæmorrhage that takes place during the operation at least one-half, and also prevents in part the formation of any croupous exudation over the cut surface. If the quinine as given above does not remove the congested appearance of the turbinated bodies that it is so common to find when the septum is deflected, he augments the dose by a drachm of the Warburg's tincture twice or three times a day after eating. This preparatory treatment is very important, and upon it depends greatly the comfort of the patient after the operation. He finds the accustomed frontal headache is done away with, the liability to secondary hæmorrhage is diminished, and a clearer view of the offending obstruction during the time of the operation is secured. Thus, the saw can be guided more thoroughly, and the protruding deflection removed thoroughly at one sitting. Before operating, he sprays into the nose a 2 per cent. solution of cocaine. After waiting a few moments for this to act, he then applies by means of a probe wrapped with cotton, a 20 per cent. solution of cocaine. When the mucous membrane is thoroughly anæsthetised, Griffin introduces the saw and makes his cut from below upward; this allows him to guide his saw upon tissue that is not covered with blood, and to perceive how much of the septum it is necessary to pare off to produce normal breathing space. Where the septum is broad at its base Griffin makes what he calls an incut. This is accomplished by carrying the blade of the saw parallel with the floor of the nose into the obstruction and then turning it upward. This makes a clean cut and leaves a perfectly symmetrical septum at the base. This can only be done in cases where the septum is greatly thickened, and must be done with discrimination, otherwise more of the septum is removed than was indicated, and a perforation is liable to be the sequel.

After the septum has been completely sawed through the snare is passed over the protruding part and cuts the mucous membrane that sometimes holds it at its superior surface. After the piece is removed he administers from six to ten grains of quinine to the patient with perhaps ten to fifteen grains of the bromide of potassium. This generally prevents any secondary hæmorrhage. The hæmorrhage from the operation only lasts a few moments in the majority of cases, and this is especially true if the case has been properly prepared beforehand, and the case is not of the hæmorrhagic diathesis. A secondary hæmorrhage of no moment is liable to occur from one to three hours after the operation. Griffin wards this off by putting a small plug of cotton into the nose, and directs his patient to remove it about five hours from the time of the surgical procedure. The quicker the plug is removed the better it is, as it prevents a free drainage, and if retained for any length of time will produce an inflammation and give rise to an elevation of temperature and concomitant headache. The author uses no dressing whatsoever to the cut surface. He allows it to scab over, and let Nature heal it under this protection. Salves retard the healing by removing the crusts sooner than indicated; but he allows a wash at times if the nose be much stenosed, but this is an unusual sequence if the preparatory treatment above outlined has been followed out. The quinine is given for one to three weeks after the operation, Warburg's tincture when necessary.

By these means of procedure Griffin has been able to operate in over *three thousand cases* of deflected septum, and he can enumerate only three cases that have given any concern in regard to hæmorrhage following the operation. If the cut has been clean, the cut surface of the septum heals over entirely in about a month, and the patient has established a patency of the nose, the object sought by the operation.

[11] Med. and Surg. Rep., July 25; Journ. of Laryngology, Mar., 1897.
[12] New York Med. Journ., Jan. 16, 1897. [13] Med Week, Oct. 9, 1896.
[14] New York Med. Journ, June 12, 1897.

